# Intranasal Leukemia Inhibitory Factor Attenuates Gliosis and Axonal Injury and Improves Sensorimotor Function After a Mild Pediatric Traumatic Brain Injury

**DOI:** 10.1089/neur.2021.0075

**Published:** 2023-04-11

**Authors:** Veera D'Mello, Malini Subramaniam, Aditya Paul Bhalla, Sherlyn Saavedra, Ofri Leiba, Steven W. Levison

**Affiliations:** Department of Pharmacology, Physiology, and Neuroscience, New Jersey Medical School, Rutgers University, Newark, New Jersey, USA.

**Keywords:** astrogliosis, blood–brain barrier, microgliosis, neurotrauma, traumatic axonal injury

## Abstract

Leukemia inhibitory factor (LIF) is a neuroprotective cytokine that is essential for appropriate glial responses, remyelination, and preservation of neuronal conductance after injury. The intranasal route for delivery of therapeutics to the central nervous system is of particular interest given that it bypasses the blood–brain barrier and peripheral clearance systems. We explored the possibility that LIF might improve neurological function when administered intranasally during the acute phase in a pediatric model of mild traumatic brain injury (mTBI). We tested two doses of LIF and evaluated behavioral outcomes. Here, we show that acute 40-ng intranasal LIF treatment twice a day for 3 days attenuates astrogliosis and microgliosis, protects against axonal damage, significantly improves sensorimotor function, and is well tolerated without detrimental effects on growth. Altogether, our studies provide pre-clinical evidence for the use of acute intranasal LIF treatment as a viable therapeutic for pediatric cases of mTBIs.

## Introduction

Traumatic brain injuries (TBIs) are a significant cause of disability and death worldwide in the civilian population. In the United States alone, it is estimated that at least 1.7 million people suffer a TBI each year, resulting in 235,000 hospitalizations and 50,000 deaths.^1^ Moreover, 30% of TBIs occur in children under the age of 14 years.^2^ Pediatric TBIs are especially detrimental because they can alter the trajectory of brain development. Whereas it has been thought that a single concussive hit to the head does not produce long-term behavioral effects, approximately one fifth of mild TBIs (mTBI) produce long-term deficits, including poor memory, reduced processing speed, fatigue, impulsivity, anxiety, or depression.^3^ Progress is being made in developing neuroprotective strategies to reduce acute brain injury,^4–6^ and several U.S. Food and Drug Administration–approved drugs are being investigated^7,8^; however, clinical trials are still in the early stages.

The cytokine, leukemia inhibitory factor (LIF), has several remarkable properties that recommend pursuing it as a therapeutic. LIF has the potential to reduce neuronal cell death, mobilize stem cells within the brain, increase oligodendrocyte production and maturation, and improve neurological function. In a previous study, we found that LIF haplodeficiency desynchronizes microgliosis and astrogliosis accompanied by increased neurodegeneration after a closed head injury.^9^ Subsequently, using a neonatal hypoxia-ischemia injury model, we demonstrated that providing LIF intranasally (IN) 3 days after injury provided remarkable neuroprotection as measured histologically and using behavioral assays.^10^ Similarly, in a study using a mouse model of multiple sclerosis, a high dose of LIF (25 μg/kg/d) slowed the progression of disease when administered for 18 days beginning at the induction of disease. In a subsequent experiment, the cohort that received LIF after disease symptoms were evident had significantly reduced disease scores. Further, by day 24, 80% of placebo animals had died, compared with 45% of LIF-treated (Rx) animals.^11^ Similarly, promising results have been reported using a different model of multiple sclerosis as well as in a mouse model of spinal cord injury.^12,13^ Importantly, in a clinical trial, recombinant human LIF, under the trade name of Emfilermin, was tested in a randomized, double-blinded, placebo-controlled phase II trial for chemotherapy-induced peripheral neuropathy. LIF was well tolerated with 95% compliance and no adverse effects on quality of life; however, there were no differences between groups on any of the neurological tests performed. Here, we tested the efficacy of IN-LIF when administered 4 h after an mTBI in post-natal day 18 (P18) mice.

## Methods

### Reagents

Common laboratory chemicals were purchased from either Sigma-Aldrich (St. Louis, MO) or VWR (Radnor, PA). Recombinant murine LIF was purchased from Millipore (catalog no.: LIF2010). Antibodies, their source, and dilutions are listed in Table 1. Western lightning chemiluminescence reagent was obtained from PerkinElmer (Wellesley, MA).

**Table 1.** List of Antibodies Used to Detect Proteins by Immunofluorescent Staining *in situ* or by Western Blotting

Antibody	Host	Application and dilution	Source	Catalog no.
Glial fibrillary acidic protein (GFAP)	Rabbit	Immunofluorescence, 1:200; Western blot, 1:1000	Dako, Santa Clara, CA	Z0334
Myelin basic protein (MBP)	Rabbit	Immunofluorescence, 1:200; Western blot, 1:1000	Abcam, Cambridge, MA	ab40390
Amyloid precursor protein (APP) Y188	Rabbit	Immunofluorescence, 1:200	Abcam, Cambridge, MA	ab32136
Neurofilament H (NF-H) phosphorylated: SMI-31	Mouse	Immunofluorescence, 1:200	BioLegend, San Diego, CA	801601
Ionized calcium-binding adaptor molecule 1 (Iba1)	Rabbit	Immunofluorescence, 1:200	Wako Pure Chemical Corporation, Wako, TX	019-19741
CD68	Rat	Immunofluorescence, 1:200; Western blot, 1:1000	Bio-Rad, San Diego, CA	MCA1957GA
Tubulin cleaved by caspase-6 (C6T)	Rabbit	Western blot, 1:1000	MediMabs, Montreal (Quebec) Canada	MM-0143
Claudin-5	Rabbit	Western blot, 1:1000	Abcam, Cambridge, MA	ab15106
N-cadherin	Rabbit	Western blot, 1:1000	Cell Signaling Technology, Danvers, MA	13116
Aquaporin-4	Rabbit	Western blot, 1:1000	ThermoFisher Scientific, Waltham, MA	PA5-78812
Actin	Mouse	Western blot, 1:1000	Sigma-Aldrich, St. Louis, MO	A5441
Anti-mouse IgG-HRP	Donkey	Western blot, 1:2500	Jackson ImmunoResearch, West Grove, PA	715-035-150
Anti-rabbit IgG-HRP	Donkey	Western blot, 1:2500	Jackson ImmunoResearch, West Grove, PA	711-035-152
RedX-conjugated anti-mouse IgG	Donkey	Immunofluorescence, 1:300	Jackson ImmunoResearch, West Grove, PA	715-295-150
Alexa 488–conjugated anti-rabbit IgG	Donkey	Immunofluorescence, 1:300	Jackson ImmunoResearch, West Grove, PA	711-545-152
Alexa 594–conjugated anti-rabbit IgG	Donkey	Immunofluorescence, 1:300	Jackson ImmunoResearch, West Grove, PA	711-585-152
Alexa 488–conjugated donkey anti-mouse IgG	Donkey	Immunofluorescence, 1:300	Jackson ImmunoResearch, West Grove, PA	715-545-150

CD68, cluster of differentiation 68; IgG, immunoglobulin G; HRP, horseradish peroxidase.

### Rodents

All experiments were approved by the Institutional Animal Care and Use Committee of Rutgers New Jersey Medical School (Protocol No.: 999900841) and were in accordance with the National Institutes of Health (NIH) Guide for the Care and Use of Laboratory Animals (NIH publication no.: 80-23, revised in 1996) and ARRIVE (Animal Research: Reporting of In Vivo Experiments) guidelines. Wild-type (WT) adult CD1 mice were purchased from Charles River Laboratories (Wilmington, MA) to create a breeding colony that was maintained in our vivarium. Animals were group housed and kept on a 12-h light/dark cycle with *ad libitum* access to food and autoclaved water. Pups remained with the dam until euthanasia.

### Mild traumatic brain injury

**FIG. 1.**
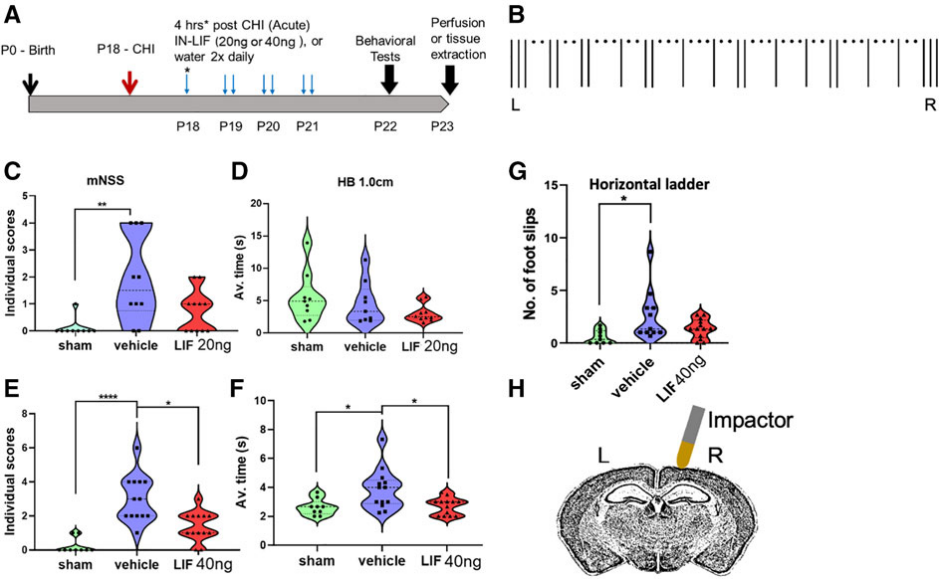
Acutely administered IN-LIF improves sensorimotor function 5 days after a pediatric closed head injury. (**A**) Schematic of experimental design. CD1 wild-type (WT) mice were subjected to mTBI on P18. IN-LIF (2 μg/mL) was administered as either a 10-μL (20-ng) or 20-μL (40-ng) dose acutely 4 h after mTBI and every 12 h thereafter two times daily for 3 consecutive days (P19–P21). A matched volume of double distilled water was administered as vehicle control. Behavioral tests were performed on the fourth day at P22. Mice were euthanized for tissue processing on the fifth day at P23. (**B**) Pattern of horizontal ladder used to quantify fine motor function. Mice were placed on the left (L) end of the ladder and motivated to walk to the right (R) end by placing a food pellet on the landing at the R-end. (**C,E**) Modified Neurological Severity Scores (mNSS) of sham, IN-vehicle-, and IN-LIF-treated mice (*p < 0.05, **p < 0.005, *n* = 9–11, ****p < 0.0001, *n* = 9–13, by Kruskal-Wallis' test followed by Dunn's multiple comparisons test). (**D,F**) Average time required to traverse an elevated 1.0-cm horizontal beam (HB; *p < 0.05; *F*_(2, 32)_ = 6.147, *p* = 0.0055, *n* = 9–13, by one-way ANOVA followed by Tukey's multiple comparison test). (**G**) Average number of footslips of three non-consecutive runs on horizontal ladder (*p < 0.05, *n* = 9–11, *F*_(2, 30)_ = 4.561, *p* = 0.0186, by one-way ANOVA followed by Tukey's multiple comparison test). (**H**) Schematic figure of coronal view of the injury location approximately at −2.0 bregma. A pneumatic metal impactor was driven at a 5-degree angle directly onto the exposed skull on the right hemisphere (R). ANOVA, analysis of variance; CHI, closed head injury; LIF, leukemia inhibitory factor; IN-LIF, intranasal LIF .

WT male and female CD1 mice received a single closed TBI on P18, an age where their brains represent those of humans 2–3 years of age.^14^ Briefly, after being administered 0.05 mg/kg of buprenorphine, mice were anesthetized with isofluorane, an incision was made along the midline, and the scalp was deflected and then subjected to mTBI using a sterile 5-mm rounded metal impactor tip positioned at a 5-degree angle onto the right side of the sagittal suture halfway between the bregma and lambda. The impactor accelerated to the skull at a velocity of 4 m/sec, to a depth of 1.0 mm past zero point on the skull surface, with a dwell time of 150 msec (Custom Design & Fabrication eCCI Model 6.3, catalogue no.: 23298-047; Custom Design & Fabrication, Inc., Sandston, VA; Fig. 1H). These impact parameters result in injury that encompasses the retrosplenial cortex, primary somatosensory area and motor area, and adjacent primary motor cortex, producing measurable gross motor dysfunction. Age-matched, sham-operated animals were anesthetized and received only a scalp incision. Given that the skull of a P18 mouse is pliable, no skull fractures were observed in any of the injured mice. Hematoxylin-eosin–stained coronal sections in the area of injury around −2.5 bregma did not show any overt morphological changes (data not shown).

### Leukemia inhibitory factor administration

An acute dose of 20 or 40 ng of recombinant murine LIF at 2 μg/mL (#LIF2010; MilliporeSigma, Burlington, MA), dissolved in water, was administered as 2-μL drops alternately to each nostril, every 2 min while mice were anesthetized with isoflurane. The first dose of LIF was given 4 h post-injury and then every 12 h for 3 days. For subacute administration, 40 ng of LIF was administered, as described above, beginning 72 h post-injury every 12 h for 3 days.

### Behavioral tests

Animals were handled for 2 days before administering the tests. Post-traumatic neurological impairments were assessed using a 12-point modified Neurological Severity Score (mNSS; Table 2). Animals were tested at 4 days post-injury (dpi; P22). High final mNSS scores were indicative of task failures. Gross motor function was assessed by measuring average time taken to traverse an elevated 1.0-cm horizontal beam. Fine motor skills were analyzed on a horizontal ladder with rungs spaced unevenly to increase the complexity of the run (Fig. 1B). Average numbers of footslips over three non-consecutive runs were calculated for each animal. All beam tasks and horizontal ladder walk tests were video recorded and analyzed in a blinded fashion. Mice from two to three litters were assessed for each group.

**Table 2.** Modified Neurological Severity Score

Task	Description	Success	Failure
Exit circle	Ability and initiative to exit a circle of 30 cm diameter (time limit = mean + 2 SDs of time taken by sham mice)	0	1
Mono-/hemiparesis	Paresis of upper and/or lower limb	0	1
Straight walk	Alertness, initiative, and motor ability to walk straight	0	1
Tail position	Tail position is either up (normal) or down (impaired) while walking	0	1
Startle reflex	Innate reflex; the mouse bounces in response to a loud hand clap	0	1
Seeking behavior	Physiological behavior as a sign of “interest” in the environment	0	1
Grip test	Ability to grip forceps with all four limbs	0	1
Beam balancing	Ability to balance on a beam of 7 mm width for at least 10 sec	0	1
Round stick balancing	Ability to balance on a round stick of 5 mm diameter for at least 10 sec	0	1
Beam walk: 3 cm	Ability to cross a 30-cm-long beam of 3 cm width (time limit = mean + 2 SDs of time taken by sham mice)	0	1
Beam walk: 2 cm	Same task, increased difficulty on a 2-cm-wide beam	0	1
Beam walk: 1 cm	Same task, increased difficulty on a 1-cm-wide beam	0	1
**Maximal score**			**12**

Each mouse was administered a series of 12 tests. Successful completion of a test was scored 0, and a failed test was scored 1. Total score was computed by adding scores of all 12 tests for each animal. Higher scores indicate greater impairment.

SDs, standard deviations.

### Western blot analyses

At 4 dpi, mice were heavily dosed with ketamine/xylazine (90/10 mg/kg) and then decapitated. The retrosplenial cortex and corpus callosum (CC) below the impactor (−1.0 to −3.0 mm bregma) was collected and flash frozen on dry ice. Then, 20 μg of protein was separated by sodium dodecyl sulfate/polyacrylamide gel electrophoresis, and western blots were performed as previously described,^15^ using the antibodies listed in Table 1. Imaging was performed using a BioRad ChemiDoc Imaging System combined with Image Lab software (Bio-Rad Laboratories, Hercules, CA). Band intensity for each protein was normalized against β-actin levels.

### Intracardiac perfusions and sectioning

Mice that had received LIF acutely (4 h after mTBI) were terminated 2 days after the final LIF dose (P23) by intracardiac perfusion, and brains were sectioned at 20 μm.^10^ Mice that had received LIF subacutely (starting on P21, 3 dpi) were terminated on P26. Consecutive sections were collected beginning −1.5 mm bregma to −2.5 mm bregma, which spanned the area directly under the point of impact. Sections were mounted onto SuperfrostPlus slides (VWR) and stored at −30°C.

### Histology and immunofluorescence

Immunofluorescence was performed as previously described,^9^ with the exception of the staining method for myelin basic protein (MBP) where sections were delipidated for 10 min in 100% ethanol before blocking. No signal above background was obtained when the primary antibodies were replaced with pre-immune sera. Images were collected using a QImaging Retiga-2000R CCD camera (QImaging, Surrey, BC, Canada) on an Olympus AX70 microscope (Olympus America Inc., Center Valley, PA). Images were analyzed using FiJi software in automated batch mode using a custom script. Briefly, to measure intensity, RGB channels were split, background was subtracted by applying rolling ball radius, and the individual channel intensity was read. For percentage area and particle count analyses, images were then further thresholded, converted to mask, and outlined. Then, the “Analyze particles” command was run defining size (0 to infinity) and circularity (0–1) that produced a summary file with particle count and percent area values. Images were randomly checked in all three groups to ensure that the set parameters did not result in false positives or false negatives. Images quantified for ionized calcium-binding adaptor molecule 1 (Iba1)-positive microglia and Iba1/CD68 (cluster of differentiation 68) double-positive microglia were coded and then manually counted.

### Statistical analyses

Raw data from image analyses and behavioral tests were imported into Prism (GraphPad Software; La Jolla, CA) for statistical analyses, using one-way analysis of variance (ANOVA) followed by Tukey's *post hoc* intergroup comparison. Graphs were produced in Prism, and error bars denote standard error of means. Comparisons were interpreted as significant when associated with *p* < 0.05. Non-parametric mNSS data were analyzed by Kruskal-Wallis' test followed by Dunn's *post hoc* test.

## Results

### Acutely administering leukemia inhibitory factor intranasally improves sensorimotor function after pediatric mild traumatic brain injury

To test the hypothesis that increasing the level of LIF early after injury might improve neurological function, we subjected P18 mice to mTBI and then infused two doses of IN-LIF: 20 and 40 ng (Fig. 1A). Neurological function was evaluated using mNSS.^9^ mTBI significantly impaired performance on mNSS in IN-vehicle Rx (Figs. 1C,E). The 20-ng IN-LIF Rx showed a trend toward improving behavioral outcomes, but did not reach statistical significance. The 40-ng IN-LIF Rx significantly reduced the average mNSS score over IN-vehicle Rx (Fig. 1E; *p < 0.05, *n* = 9–11). Similarly, 40-ng IN-LIF Rx significantly shortened beam walking time (Fig. 1F; *p < 0.05, *n* = 9–13 vs. IN-vehicle Rx). On the horizontal ladder (Fig. 1B), the IN-vehicle group had significantly more footslips than sham animals (*p < 0.05, *n* = 9–11). The 40-ng IN-LIF Rx group showed a trend toward fewer footslips over the IN-vehicle group (*p* = 0.14, *n* = 11–13) that did not differ significantly from sham animals (*p* = 0.46, *n* = 9–13; Fig. 1G, Supplementary Fig. S1A–C). Overall, 40-ng IN-LIF Rx showed better efficacy; therefore, for all subsequent studies, we compared 40-ng IN-LIF Rx post-injury to vehicle Rx and sham-operated mice. We did not observe an effect of sex on LIF Rx or vehicle Rx using the mNSS test at P22 (Supplementary Fig. S1D). We therefore combined male and female mice for all analyses in this study.

### Intranasal/leukemia inhibitory factor attenuates astrogliosis in the injured neocortex after pediatric mild traumatic brain injury

**FIG. 2.**
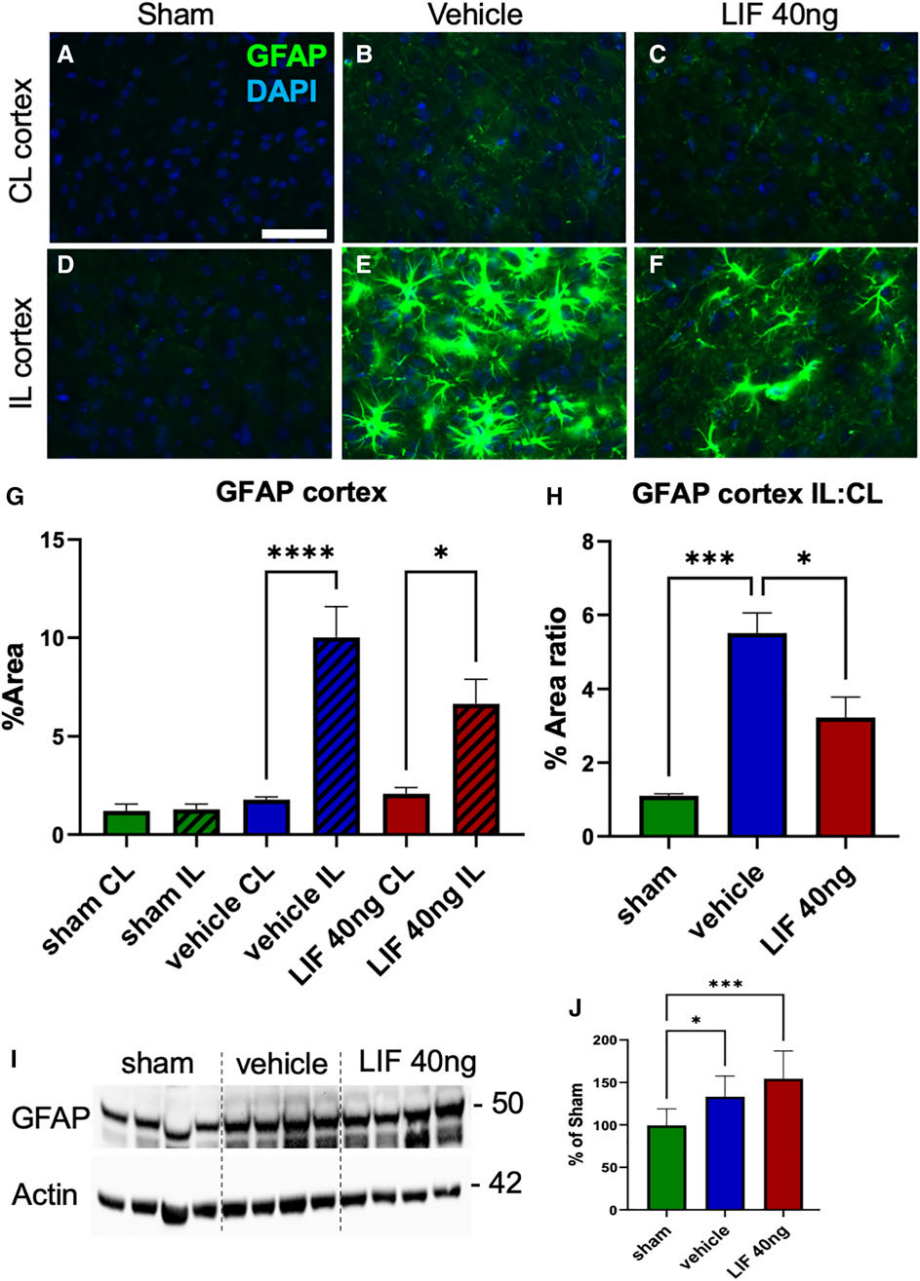
Acutely administered IN-LIF attenuates astrogliosis in the neocortex 5 days after a pediatric closed head injury. (**A–F**) Representative images of activated astrocytes as revealed by GFAP immunostaining of the contralateral (CL) neocortex (**A–C**) versus ipsilateral (IL) neocortex (**D–F**) for sham (**A,D**), IN-vehicle-treated (**B,E**), and 40-ng IN-LIF-treated (**C,F**) mice. (**G**) Comparison of percent area per field of view (FOV) for GFAP expression (*p < 0.05, ****p < 0.0001; *F*_(5, 18)_ = 18.71, *p* < 0.0001, *n* = 4). (**H**) Percent area of GFAP expression in IL versus CL neocortices (*p < 0.05, ***p < 0.0005; *F*_(2, 9)_ = 24.19; *p* = 0.0002, *n* = 4). (**I,J**) Western blot analysis of the IL neocortex and underlying corpus callosum (*p < 0.05, ***p < 0.001; *F*_(2, 25)_ = 9.671, *p* = 0.0008; *n* = 8–10, by one-way ANOVA followed by Tukey's multiple comparison test). Scale bar = 50 μm. ANOVA, analysis of variance; mTBI, closed head injury; DAPI, 4′,6-diamidino-2-phenylindole; GFAP, glial fibrillary acidic protein; LIF, leukemia inhibitory factor; IN-LIF, intranasal LIF.

In our study of mTBI in LIF heterozygous mice, we showed that, over time, there was an exaggerated astroglial response to injury.^9^ Therefore, we next evaluated the effect of 40-ng IN-LIF Rx on glial fibrillary acidic protein (GFAP) expression. Contralateral (CL) neocortices of sham, IN-vehicle, and IN-LIF Rx groups showed negligible GFAP immunofluorescence (Figs. 2A–C). By contrast, hypertrophic GFAP^+^ astrocytes were prominent in the ipsilateral (IL) neocortex of IN-vehicle Rx animals (Fig. 2E) and occupied a significantly greater percentage of area per field of view (FOV) than in the CL neocortex (Fig. 2G; *p* < 0.0001). The 40-ng IN-LIF Rx reduced the number as well as area of GFAP^+^ astrocytes in the IL neocortex (Fig. 2F,G), although GFAP staining in the IL neocortex of IN-LIF Rx animals was significantly higher than the CL neocortex (Fig. 2E,B,G; *p* < 0.05). Comparing the ratio of GFAP between the IL and CL neocortices, there was an increased ratio in the IN-vehicle group compared to sham (Fig. 2H; *p* < 0.0005) that was significantly reduced after IN-LIF Rx (Fig. 2H; *p* < 0.05 vs. IN-vehicle). GFAP staining in the CC did not differ between groups (Supplementary Fig. S2).

Western blot analysis of neocortical tissue, including overlying the CC, showed increased GFAP expression in the injured groups over sham. However, IN-LIF Rx did not reduce GFAP levels compared to IN-vehicle Rx animals (Fig. 2I,J). Taken altogether, these data show that 40-ng IN-LIF Rx attenuates the astroglial response to pediatric TBI.

### Intranasal/leukemia inhibitory factor attenuates macrophage/microglial activation in the injured neocortex after pediatric mild traumatic brain injury

**FIG. 3.**
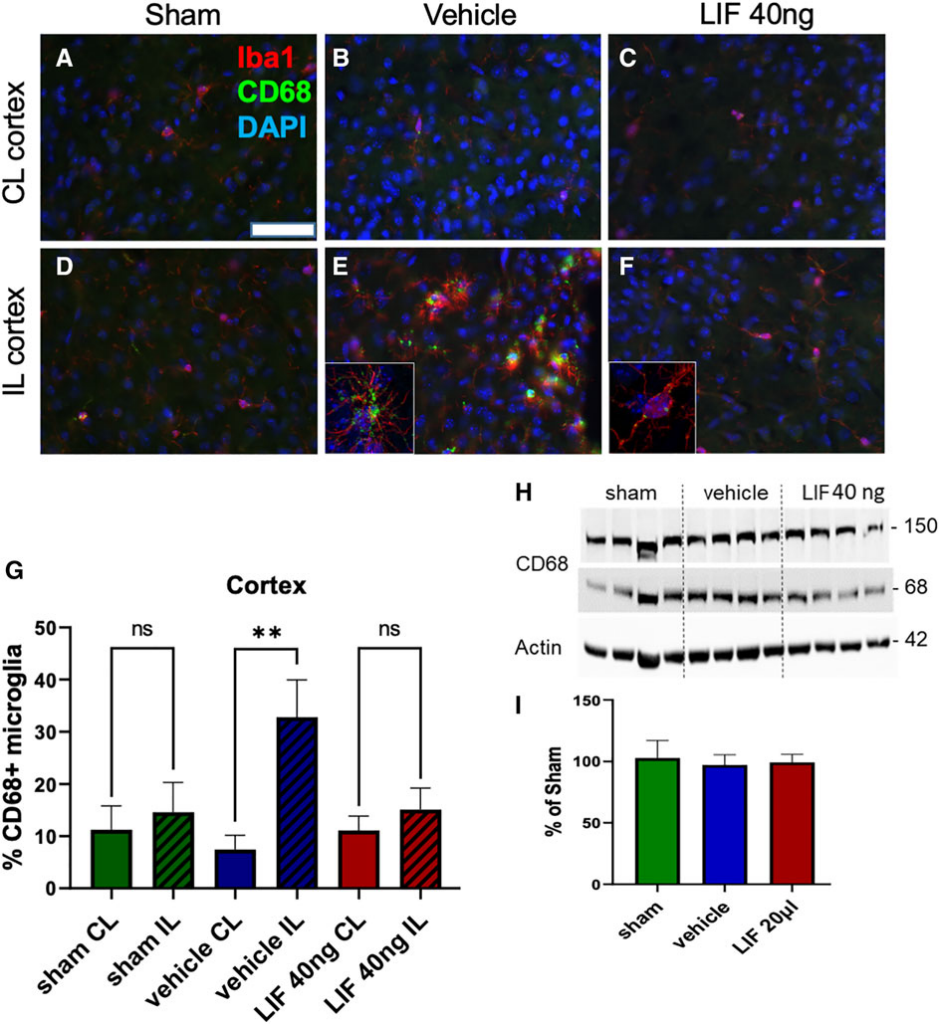
Acutely administered IN-LIF attenuates microgliosis and phagocytic activity in the neocortex 5 days after a pediatric closed head injury. (**A–F**) Representative images of activated microglia as revealed by coimmunostaining for Iba1 and the lysosomal protein CD68 in the contralateral (CL) neocortex (**A–C**) and ipsilateral (IL) neocortex (**D–F**) for sham (**A,D**), IN-vehicle-treated (**B,E**), and 40-ng IN-LIF-treated (**C,F**) mice. Insets in (**E**) and (**F**) are magnified images of phagocytosing microglia. (**G**) Percent Iba1/CD68 double-positive microglia between hemispheres for sham-operated, IN-vehicle-, and IN-LIF-treated mice (**p < 0.01; *F*_(5, 36)_ = 3.476, *p* = 0.0115, *n* = 6–9). (**H,I**) Western blot analysis of the IL neocortex and underlying corpus callosum. CD68 levels were normalized to actin, then expressed as percent of sham (*n* = 5–9 per group). Data were analyzed by one-way ANOVA followed by Tukey's multiple comparison test. Scale bar = 50 μm. ANOVA, analysis of variance; CD68, cluster of differentiation 68; DAPI, 4′,6-diamidino-2-phenylindole; Iba1, ionized calcium-binding adaptor molecule 1; LIF, leukemia inhibitory factor; IN-LIF, intranasal LIF.

In our studies of TBI in LIF heterozygous mice, we observed amplified microgliosis after injury.^9^ Therefore, to test the hypothesis that IN-LIF would repress microgliosis, we coimmunostained coronal sections for IBA1 and the marker for activated microglia, CD68. CL cortices of sham and injured groups were populated by ramified IBA1^+^ cells with low levels of CD68^+^ puncta (Fig. 3A–C). The IL neocortex of IN-vehicle Rx animals, by contrast, contained clusters of hypertrophied IBA1^+^ cells that were colabeled with larger, more numerous CD68^+^ puncta, indicative of more actively phagocytosing cells (**p < 0.01; Fig. 3E and insert, Fig. 3G). Most of the microglia in 40-ng IN-LIF Rx brains had a ramified morphology, with the percentage of CD68^+^ microglia similar to sham (Fig. 3F and insert, Fig. 3G). Given that the percentage of CD68^+^ microglia in the CC did not differ between hemispheres, we combined values from contra- and ipsilateral CC for analysis. There was an increased trend in mean percentage of CD68^+^ microglia in the vehicle Rx group versus sham, but not the LIF Rx group versus sham (Supplementary Fig. S3). Western blot analysis of neocortical tissue, including the underlying CC, did not detect significant differences in CD68 expression between groups (Fig. 3H,I).

### Intranasal/leukemia inhibitory factor protects against axonal degeneration after pediatric mild traumatic brain injury

**FIG. 4.**
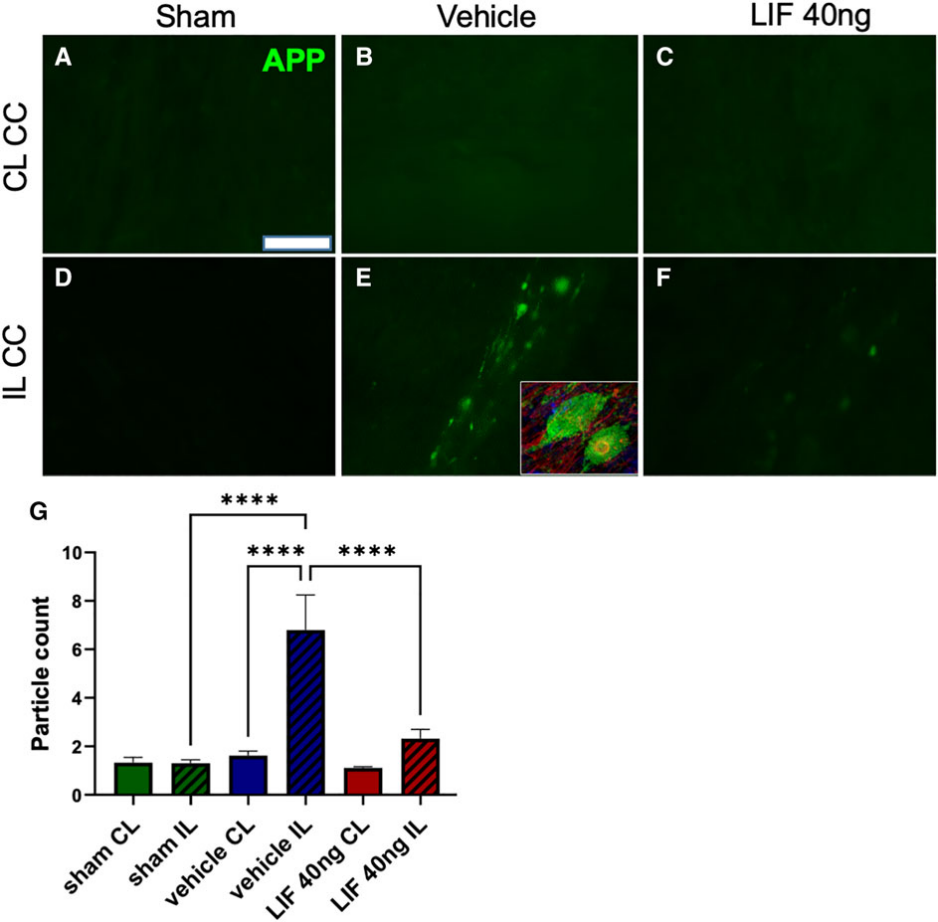
Acutely administered IN-LIF significantly decreases amyloid precursor protein (APP) accumulation in the corpus callosum (CC) 5 days after pediatric closed head injury. (**A–F**) Representative images of APP immunostaining in contralateral (CL; **A–C**) and ipsilateral (IL; **D–F**) CC fibers for sham (**A,D**), IN-vehicle-treated (**B,E**), and 40-ng IN-LIF-treated (**C,F**) mice. Inset in (**E**) shows axonal swellings containing accumulated APP (green) at higher magnification. Neurofilaments are counterstained red in the inset. (**G**) Comparison of percent area of APP accumulation in the CL and IL hemispheres (****p < 0.0001; *F*_(5, 38)_ = 12.55, *p* < 0.0001, *n* = 6–9). Data were analyzed by one-way ANOVA followed by Tukey's multiple comparison test. Scale bar = 50 μm. ANOVA, analysis of variance; LIF, leukemia inhibitory factor; IN-LIF, intranasal LIF.

Axonal transport is compromised in damaged axons, leading to the accumulation of amyloid precursor protein (APP) in axonal swellings along the length of the axon. Additionally, axons can be transected during a TBI, producing end-bulbs.^16^ Therefore, we evaluated the extent of axonal damage after pediatric mTBI by immunostaining coronal sections using the Y188 antibody that is highly specific for APP.^17^ Negligible APP staining was observed in the CL CC in all groups (Fig. 4A–D). By contrast, APP^+^ puncta accumulated within IL CC fibers of the IN-vehicle group as distinct swellings or end-bulbs (Fig. 4E). Figure 4E shows a confocal image of APP^+^ (green) within end-bulbs of severed axons in the IN-vehicle Rx group counterstained for neurofilaments (red, SMI-31 staining). The 40-ng IN-LIF Rx decreased the number of IL APP swellings and end-bulbs (Fig.4F). Particle analysis showed significantly increased numbers of APP^+^ particles in the IL CC of the IN-vehicle group over the IL CC of sham, which was almost completely reversed by IN-LIF Rx (Fig. 4G; ****p < 0.0001).

In damaged axons, caspase-6 becomes activated and cleaves alpha tubulin, causing microtubule breakdown.^18^ Therefore, we analyzed levels of alpha tubulin cleaved by caspase-6 (C6T) in the IL neocortices along with the underlying CC by western blotting. C6T levels in the IN-vehicle group did not significantly increase over sham. However, there was a strong trend toward decreased C6T expression in the IN-LIF Rx group compared to IN-vehicle (*p* = 0.058; Supplementary Fig. S4B,C), indicating possible suppression of caspase-6 activity and stabilization of microtubules. Staining with Fluoro-Jade C, a dye that labels degenerating neurons, showed increased labeling of axons in the IN-vehicle Rx group (*p* = 0.067) and a trend toward recovery in the IN-LIF Rx group (*p* = 0.31 vs. vehicle; Supplementary Fig. S4A). Altogether, these results indicate that IN-LIF Rx improves axon health and protects against axonal dysfunction and degeneration.

### Myelination of axons is relatively unaltered after pediatric mild traumatic brain injury

**FIG. 5.**
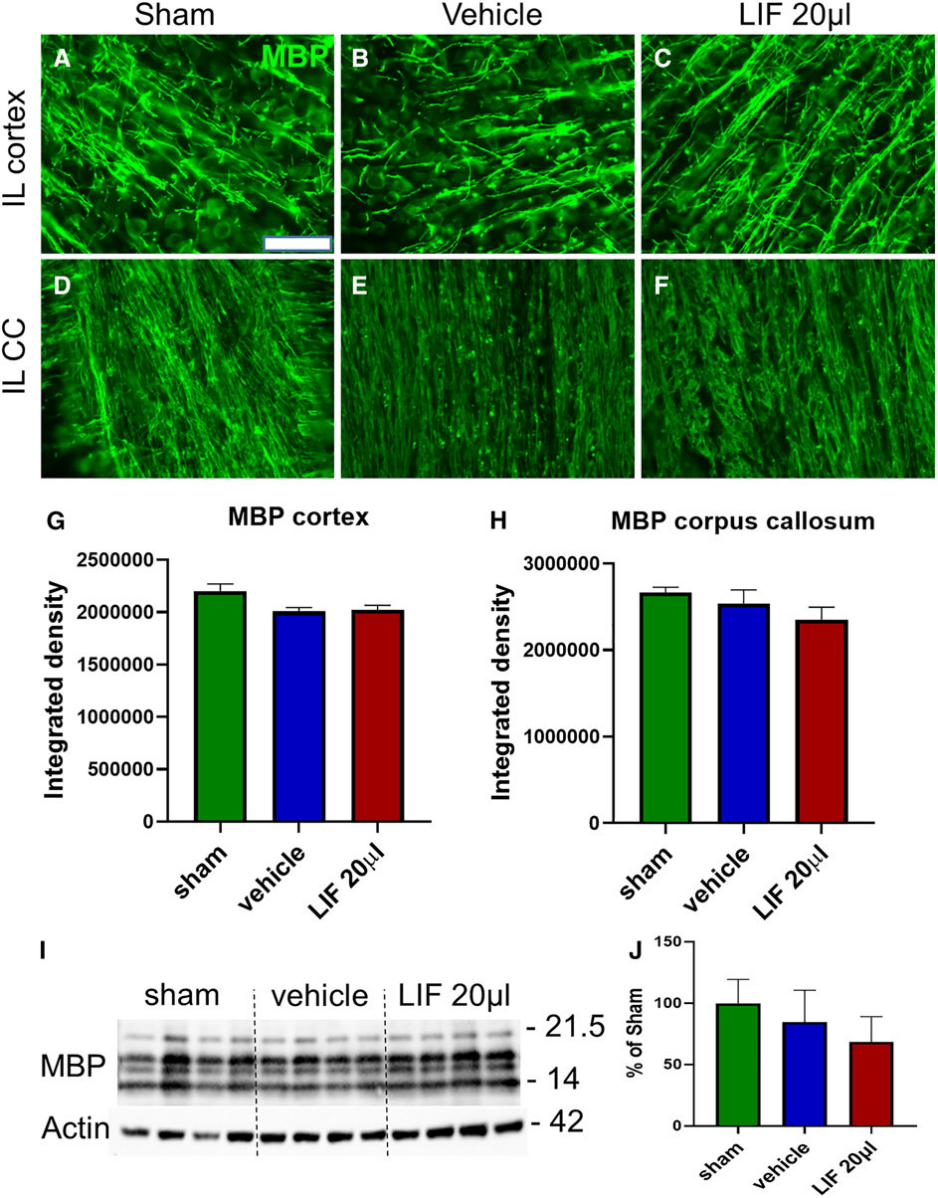
MBP levels are unaffected 5 days after pediatric closed head injury. (**A–F**) Representative images of MBP immunostaining of the ipsilateral (IL) cortex (**A–C**) and IL corpus callosum (CC; **D–F**) for sham (**A,D**), IN-vehicle-treated (**B,E**), and 40-ng IN-LIF-treated (**C,F**) mice. (**G,H**) Integrated optical density of the IL cortex (**G**) and IL CC (**H**; *n* = 4 per group). (**I,J**) Western blot analysis of MBP expression and its quantification in the injured cortex and CC (*n* = 6– 7). Data were analyzed by one-way ANOVA followed by Tukey's multiple comparison test. Scale bar = 50 μm. ANOVA, analysis of variance; LIF, leukemia inhibitory factor; IN-LIF, intranasal LIF; MBP, myelin basic protein.

To establish whether early axonal degeneration was accompanied by a loss of myelination, we immunostained coronal sections for MBP. Because there was no significant difference between expression levels in the IL or CL neocortices or CC, we combined quantitative values from IL and CL hemispheres for statistical analyses. No significant difference in integrated optical densities between groups was observed either in the neocortex or corpus callosa (Fig. 5A–H), indicating that myelination was relatively unaffected at this time point. Similarly, western blot analyses of neocortical tissue, including the underlying CC, did not show significant differences between groups (Fig. 5I,J).

### Neurovascular unit is not affected by pediatric mild traumatic brain injury

**FIG. 6.**
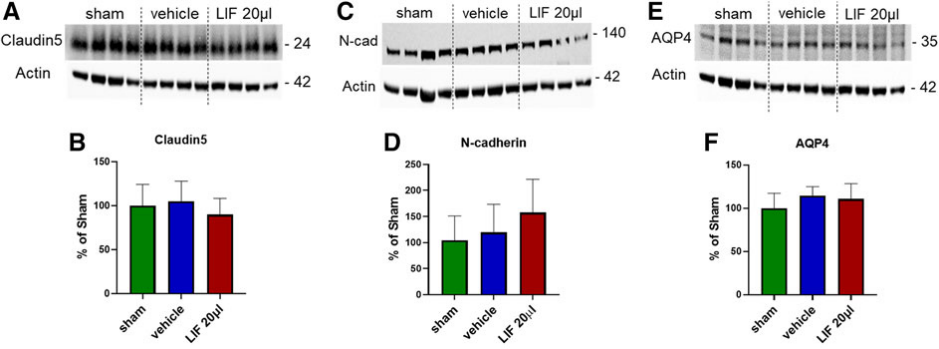
Blood–brain barrier proteins are unaltered 5 days after pediatric closed head injury. (**A,C,E**) Representative western blots of endothelial cell (EC) tight junction protein claudin-5 (**A**), EC-pericyte adhesion protein N-cadherin (**C**), and astrocyte channel protein aquaporin-4 (AQP4; **E**). (**B,D,F**) Integrated optical densities normalized to actin and expressed as the percentage of average of sham (claudin-5, *n* = 4; N-cadherin, *n* = 5–9; AQP4, *n* = 4–8). Data were analyzed by one-way ANOVA followed by Tukey's multiple comparison test. ANOVA, analysis of variance; LIF, leukemia inhibitory factor.

To determine whether blood–brain barrier (BBB) permeability was altered after a pediatric mTBI, we analyzed critical protein constituents of the BBB by western blotting. Claudin-5 forms tight junctions between adjacent endothelial cells, N-cadherin forms adherens junctions between pericytes and endothelial cells, and aquaporin-4 creates water channels at the astrocytic end-feet. TBIs perturb the BBB, leading to vasogenic edema that depends upon injury severity.^19^ Western blot analyses of BBB proteins did not show significant differences between uninjured and injured groups (Fig. 6A–F), suggesting that the neurovascular unit is relatively intact after mTBI at 5 dpi.

## Discussion

Based on our studies in LIF-haplodeficient mice,^9^ we hypothesized that increasing levels of LIF early after injury might reduce the extent of injury and preserve neurological function. Supporting this hypothesis, we show here that acutely administering LIF intranasally after mTBI depresses neocortical astrogliosis and microgliosis and reduces traumatic axonal injury, leading to improved sensorimotor recovery. Whereas a 20-ng dose showed an encouraging trend toward reducing sensorimotor dysfunction, doubling the dosage to 40 ng noticeably improved neurological function.

Given that the LIF receptor is expressed broadly throughout the brain, an important issue is whether LIF is acting directly on neurons to improve outcome, indirectly through its actions on non-neuronal cells, or whether it is acting both directly and indirectly. Here, we showed that IN-LIF reduced the numbers of APP^+^ end-bulbs and swellings in the CC. Neurons express a functional LIF receptor that promotes their survival *in vitro*.^20,21^ One mechanism through which LIF might promote neuronal survival directly is by increasing levels of the antioxidant protein, superoxide dismutase 3 (SOD3).^22,23^ Interestingly, though, in our studies of neonatal hypoxic-ischemic brain injury, IN-LIF did not increase levels of SOD3.^10^ Prokineticin-2 (ProK2) is another neurotrophic molecule that could be promoting neuronal survival in IN-LIF Rx mice. Using this mTBI model, we recently established that ProK2 messenger RNA and protein increase in the neocortex between 48 and 96 h after injury and that ProK2 induction requires intact LIF signaling.^24^ ProK2, produced by neurons, has been shown to prevent neuronal cell death by inhibiting lipid peroxidation and ferroptosis after TBI.^25^

Astrocytes play an important role in the survival and maintenance of neurons in the nervous system and are highly responsive to perturbations in brain homeostasis.^26–29^ Further, astrocyte activation after injury is deemed necessary for neuroprotection given that conditionally ablating proliferating astrocytes immediately after a TBI exacerbates the extent of neurodegeneration.^30^ Indeed, LIF-haplodeficient mice showed worse sensorimotor function beginning 2 days post-mTBI that correlated with delayed astrogliosis. Here, IN-LIF attenuated astroglial hypertrophy and decreased GFAP immunofluorescence. However, GFAP levels were unattenuated when assessed by western blotting. Though these results appear to be at odds, it has been well established that brain injuries and other stimuli that affect the astroglia cytoskeleton expose epitopes on GFAP that render it more readily detectable immunohistochemically without changes in GFAP content.^31,32^

In addition to modifying their activation, IN-LIF could be stimulating astrocyte maturation to promote their capacity to support injured neurons. The timing of this pediatric TBI coincides with astrocyte maturation, which, in mice, peaks during the third and fourth weeks post-natally. At P18, glutamine synthase, the glutamate transporters glutamate transporter 1 (GLT1) and glutamate aspartate transporter (GLAST), and the potassium channel inwardly rectifying potassium 4.1 (Kir4.1) were nearing adult levels whereas connexin43, connexin30, GFAP, S100 calcium-binding protein B (S100b), and aldolase C were still quite low.^33^ We posit that LIF might augment some of these functional proteins to improve network connectivity and neurological function. For example, increasing astrocyte glutamate transporter expression would prevent neuronal excitotoxicity by reducing extracellular levels of glutamate released by injured neurons.^34,35^

This scenario is reminiscent of pre-conditioning where a mild insult subsequently reduces the extent of injury induced by more severe injury, which correlates with increased expression of GFAP, glutamine synthetase, GLAST, monocarboxylate transporter-1, and ceruloplasmin.^36^ Similarly, enhanced Kir4.1 acquisition would promote astrocyte extracellular potassium uptake and siphoning to reduce neuronal excitability.^37^ Indeed, in a recent study of repetitive diffuse TBI, atypical astrocytes were observed that failed to express GFAP and downregulated glutamine synthetase, GLT1, connexin43, S100b, and Kir4.1. They were disconnected from surrounding astrocytes and strongly associated with post-traumatic epilepsy.^38^ In view of these findings, it is likely that acute IN-LIF Rx is accelerating the maturation of astrocytes, leading to earlier resolution of inflammation and less neuronal cell death.

Microglial cells become activated rapidly after brain injuries and produce chemokines that attract macrophages, which are generally viewed as contributing to secondary and tertiary brain injury.^39,40^ In our previous study, we found that there was a progressive accumulation of reactive microglia/macrophages in LIF-haplodeficient mice after mTBI.^9^ By contrast, we show here that IN-LIF reduces microglia hypertrophy and that the microglia are less activated as assessed by decreased expression of the lysosomal protein, CD68. These results suggest that LIF is dampening the reactivity of these cells, which should lead to reduced macrophage recruitment and ultimately less neurotoxicity. Similar to our results, systemically administered LIF has been shown to reduce numbers of activated macrophage/microglia in the ischemic rat brain, and LIF has been shown to reduce macrophage IL-12 levels *in vitro*.^41^ However, LIF does not always inhibit neuroinflammation. For example, overexpressing LIF in the mouse spinal cord stimulated macrophage/microglial proliferation that was accompanied by severe hindlimb paralysis and weight loss.^42^ We did not observe weight loss after 3 days of LIF Rx, indicating that the dosing regimen of 40 ng of IN-LIF was well tolerated (Supplementary Fig. S5).

Whereas mTBI damages the white matter resulting in demyelination, we did not observe significant changes in myelination at the early time point evaluated here. However, the beneficial effects of LIF on both immature and mature oligodendrocytes are well documented. Systemically delivered LIF promotes the functional recovery of oligodendrocytes from stroke by peroxiredoxin IV,^43^ protects oligodendrocytes against proinflammatory cytokine-induced apoptosis by inducing the expression of the antiapoptotic protein 14-3-3,^44^ and protects oligodendrocytes in a murine experimental autoimmune encephalomyelitis model independent of inflammatory cytokine modulation.^11^ Oligodendrocyte precursor cell–targeted LIF nanoparticle therapy has shown promise in the lysolecithin model of demyelinating disease.^45^

To determine the time course and distribution of IN-LIF, we used pS6RP as a parallel readout for LIF activity^46^; we found, after only 30 min of administering LIF, that pS6RP immunoreactivity was increased in the olfactory bulb and widely throughout the neocortex, which colocalized with neuronal protein neuronal nuclei in a subset of neurons (Supplementary Fig. S6).

In a P7 mouse model of neonatal hypoxia-ischemia, we initiated IN-LIF Rx beginning at 72 h post-injury (P10). In this model, delayed IN-LIF Rx significantly improved histological and behavioral outcomes when assessed at post-natal day 22.^10^ To our dismay, delaying IN-LIF Rx in this model of pediatric TBI did not significantly improve scores on the mNSS or horizontal ladder (data not shown). However, with delayed IN-LIF after pediatric mTBI, we did observe a significant increase in oligodendrocyte maturation (Supplementary Fig. S7). By contrast, we did not observe any change in oligodendrocyte maturation in the hypoxia-ischemia model. These data emphasize differences in ischemia models versus TBI models and reinforce the importance of testing potential therapeutics in appropriate pre-clinical models.

## Supplementary Material

Supplemental data

Supplemental data

Supplemental data

Supplemental data

Supplemental data

Supplemental data

Supplemental data

## Abbreviations Used

ANOVA: analysis of variance
APP: amyloid precursor protein
BBB: blood–brain barrier
CC: corpus callosum
CD68: cluster of differentiation 68
CL: contralateral
dpi: days post-injury
FOV: field of view
GFAP: glial fibrillary acidic protein
GLAST: glutamate aspartate transporter
GLT1: glutamate transporter 1
Iba1: ionized calcium-binding adaptor molecule 1
IL: ipsilateral
IN: intranasal
Kir4.1: inwardly rectifying potassium 4.1
LIF: leukemia inhibitory factor
MBP: myelin basic protein
mNSS: modified Neurological Severity Score
mTBI: mild TBI
NIH: National Institutes of Health
P: post-natal day
ProK2: prokineticin-2
Rx: treatment
S100b: S100 calcium-binding protein B
SOD3: superoxide dismutase 3
TBI: traumatic brain injury
WT: wild type
