# Supplementary material for: Intranasal Leukemia Inhibitory Factor Attenuates Gliosis and Axonal Injury and Improves Sensorimotor Function After a Mild Pediatric Traumatic Brain Injury
Source: Neurotrauma Rep. 2023 Apr 11;4(1):236–50. doi: 10.1089/neur.2021.0075 (PMC10122240; doi:10.1089/neur.2021.0075)
Supplement: Supplemental data [file Suppl_FigS1.pdf]

A
B
C

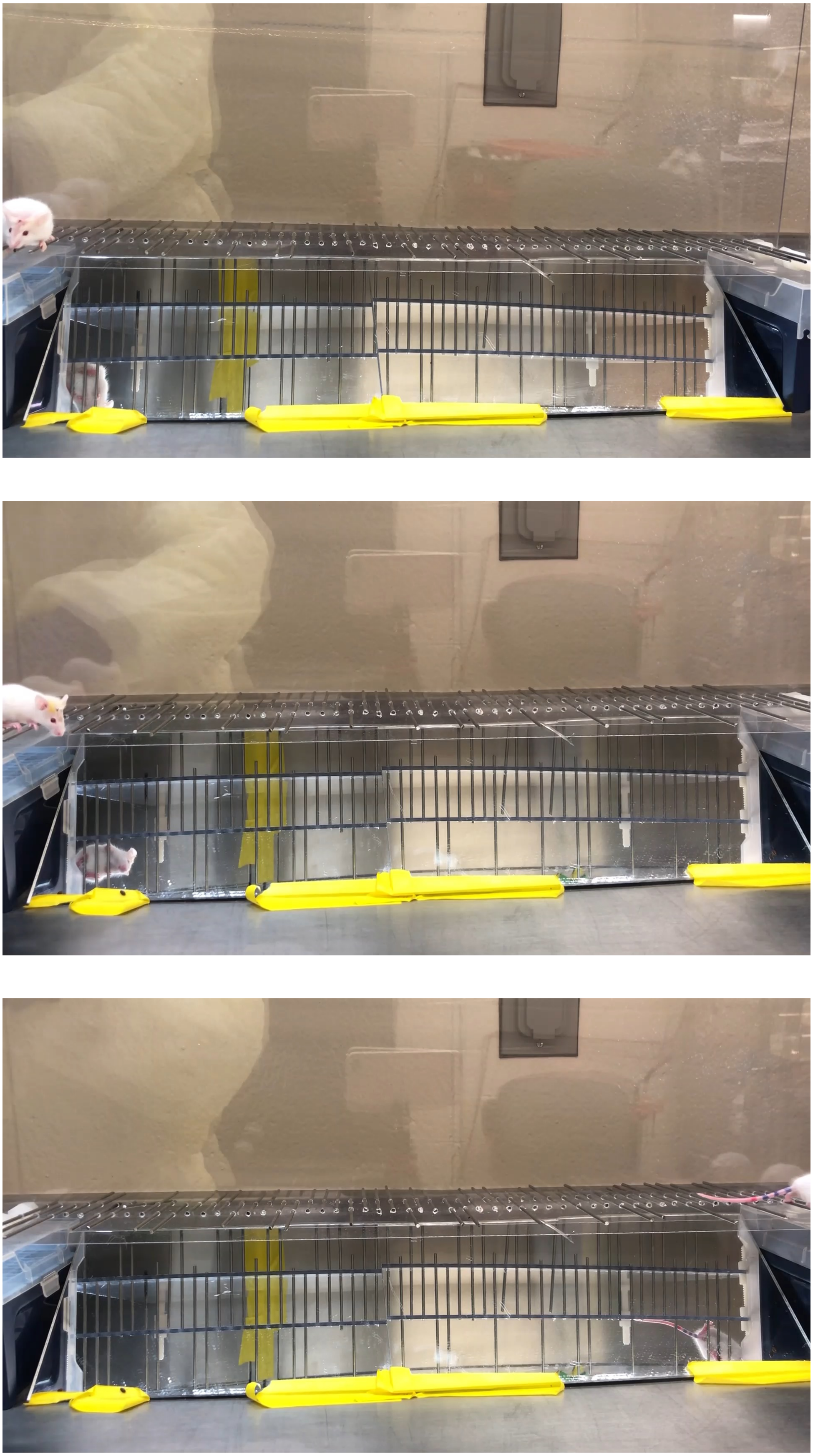

Fig S1. LIF administration decreases footslips on horizontal ladder. (A-C) Representative videos of P18 sham **(A)**, IN-vehicle Rx (B) or 40ng IN-LIF Rx **(C)** mice walking the horizontal ladder. Footslips can be observed as front or hind paws plunge below the level of rungs **(B)**.

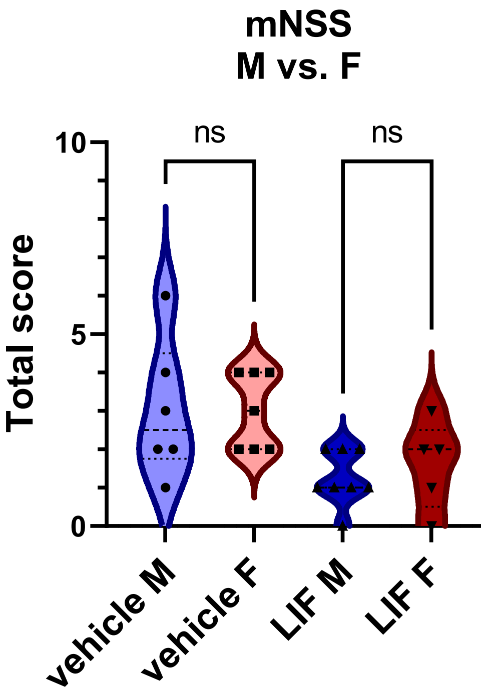

Fig S1D. Effect of LIF Rx on sensorimotor function of male and female pediatric mice. To assess the sex effect of LIF in male and female pediatric mice, mNSS tests were administered to pups at P22 after either vehicle Rx or 40ng LIF Rx. No significant differences in mNSS scores were observed between sexes in either the vehicle Rx or LIF Rx group. Data were analyzed by Kruskal-Wallis test followed by Dunn's post-hoc test. M vs. F groups: $p>0.9999$, n = 5-8 per group.
