# Supplementary material for: Intranasal Leukemia Inhibitory Factor Attenuates Gliosis and Axonal Injury and Improves Sensorimotor Function After a Mild Pediatric Traumatic Brain Injury
Source: Neurotrauma Rep. 2023 Apr 11;4(1):236–50. doi: 10.1089/neur.2021.0075 (PMC10122240; doi:10.1089/neur.2021.0075)
Supplement: Supplemental data [file Suppl_FigS2.pdf]

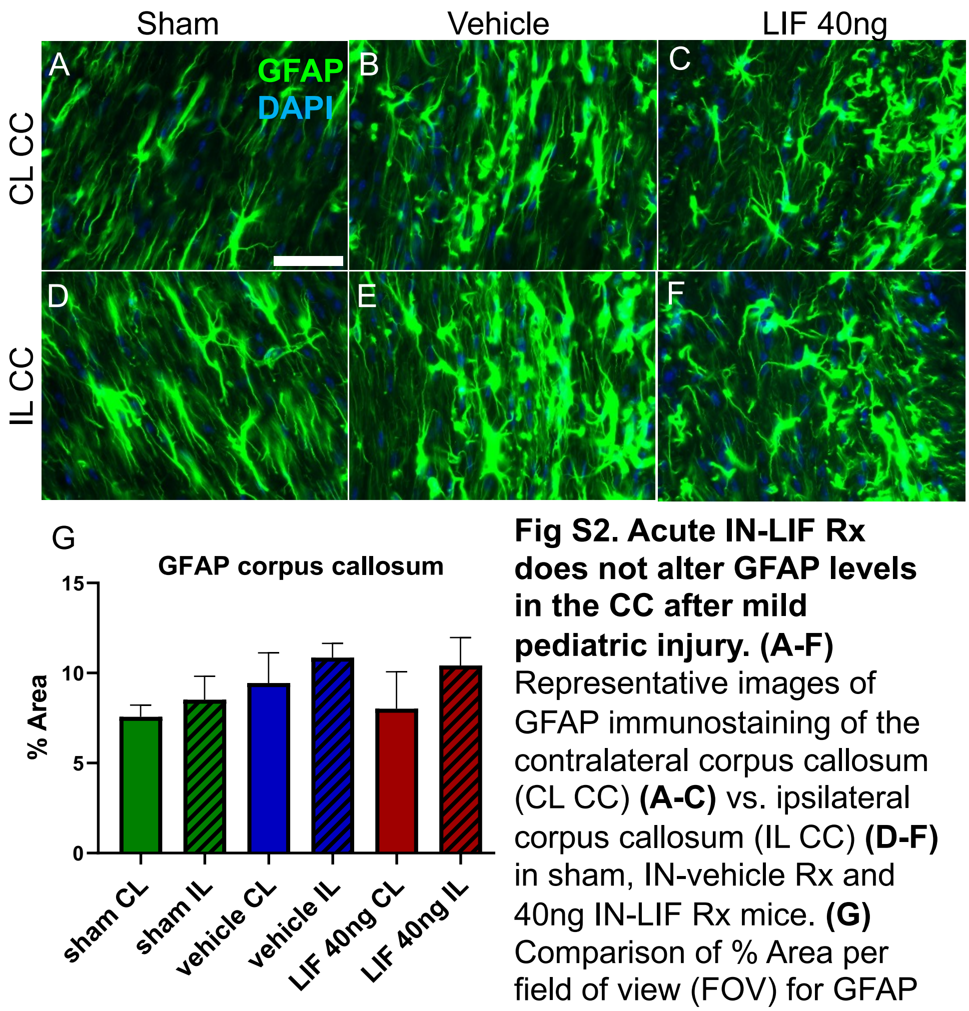

Fig S2. Acute IN-LIF Rx does not alter GFAP levels in the CC after mild pediatric injury. (A-F) Representative images of GFAP immunostaining of the contralateral corpus callosum (CL CC) **(A-C)** vs. ipsilateral corpus callosum (IL CC) **(D-F)** in sham, IN-vehicle Rx and 40ng IN-LIF Rx mice. **(G)** Comparison of % Area per field of view (FOV) for GFAP expression.
