# Supplementary material for: Intranasal Leukemia Inhibitory Factor Attenuates Gliosis and Axonal Injury and Improves Sensorimotor Function After a Mild Pediatric Traumatic Brain Injury
Source: Neurotrauma Rep. 2023 Apr 11;4(1):236–50. doi: 10.1089/neur.2021.0075 (PMC10122240; doi:10.1089/neur.2021.0075)
Supplement: Supplemental data [file Suppl_FigS3.pdf]

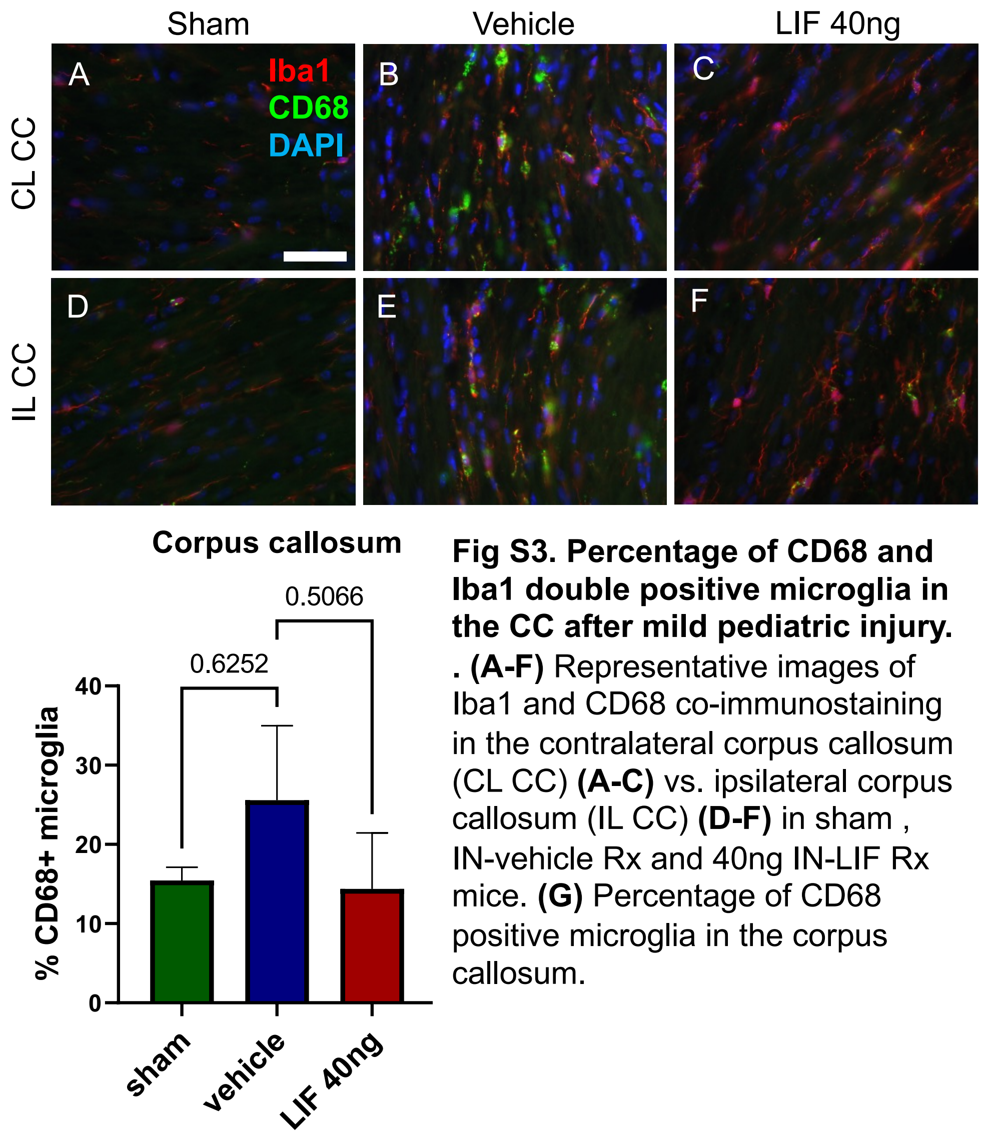

Fig S3. Percentage of CD68 and Iba1 double positive microglia in the CC after mild pediatric injury. . **(A-F)** Representative images of Iba1 and CD68 co-immunostaining in the contralateral corpus callosum (CL CC) **(A-C)** vs. ipsilateral corpus callosum (IL CC) **(D-F)** in sham , IN-vehicle Rx and 40ng IN-LIF Rx mice. **(G)** Percentage of CD68 positive microglia in the corpus callosum.
