# Supplementary material for: Intranasal Leukemia Inhibitory Factor Attenuates Gliosis and Axonal Injury and Improves Sensorimotor Function After a Mild Pediatric Traumatic Brain Injury
Source: Neurotrauma Rep. 2023 Apr 11;4(1):236–50. doi: 10.1089/neur.2021.0075 (PMC10122240; doi:10.1089/neur.2021.0075)
Supplement: Supplemental data [file Suppl_FigS4.pdf]

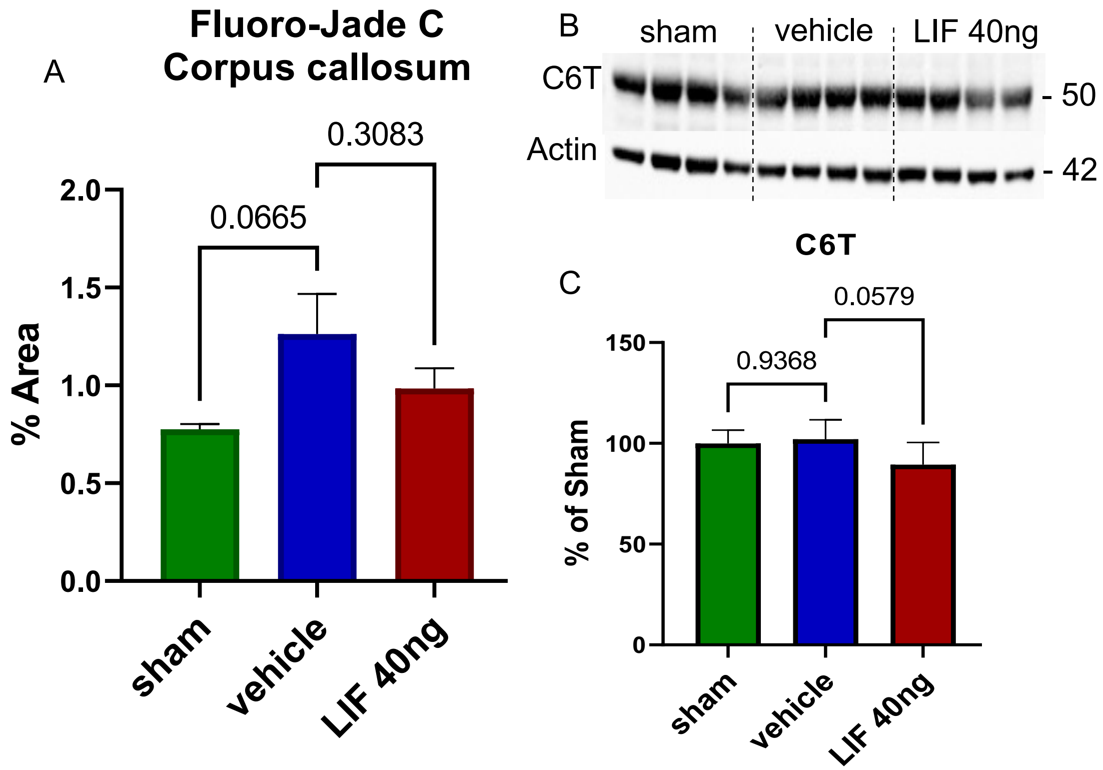

Fig S4. Markers of axonal degeneration in the corpus callosum. (A) Quantification of Fluoro-Jade C staining of the corpus callosum showed increased degeneration in vehicle Rx over sham and a trend toward recovery in LIF Rx mice (sham vs IN-LIF Rx, p = 0.53, n = 6-7 per group). **(B)** Western blot of C6T normalized against actin in brain lysates. **(C)** Quantification of western blot analysis in (B) showed decrease in tubulin cleaved by caspase 6 in IN_LIF Rx group (sham, n = 4; vehicle, n = 7; LIF, n = 8).
