# Supplementary material for: Intranasal Leukemia Inhibitory Factor Attenuates Gliosis and Axonal Injury and Improves Sensorimotor Function After a Mild Pediatric Traumatic Brain Injury
Source: Neurotrauma Rep. 2023 Apr 11;4(1):236–50. doi: 10.1089/neur.2021.0075 (PMC10122240; doi:10.1089/neur.2021.0075)
Supplement: Supplemental data [file Suppl_FigS5.pdf]

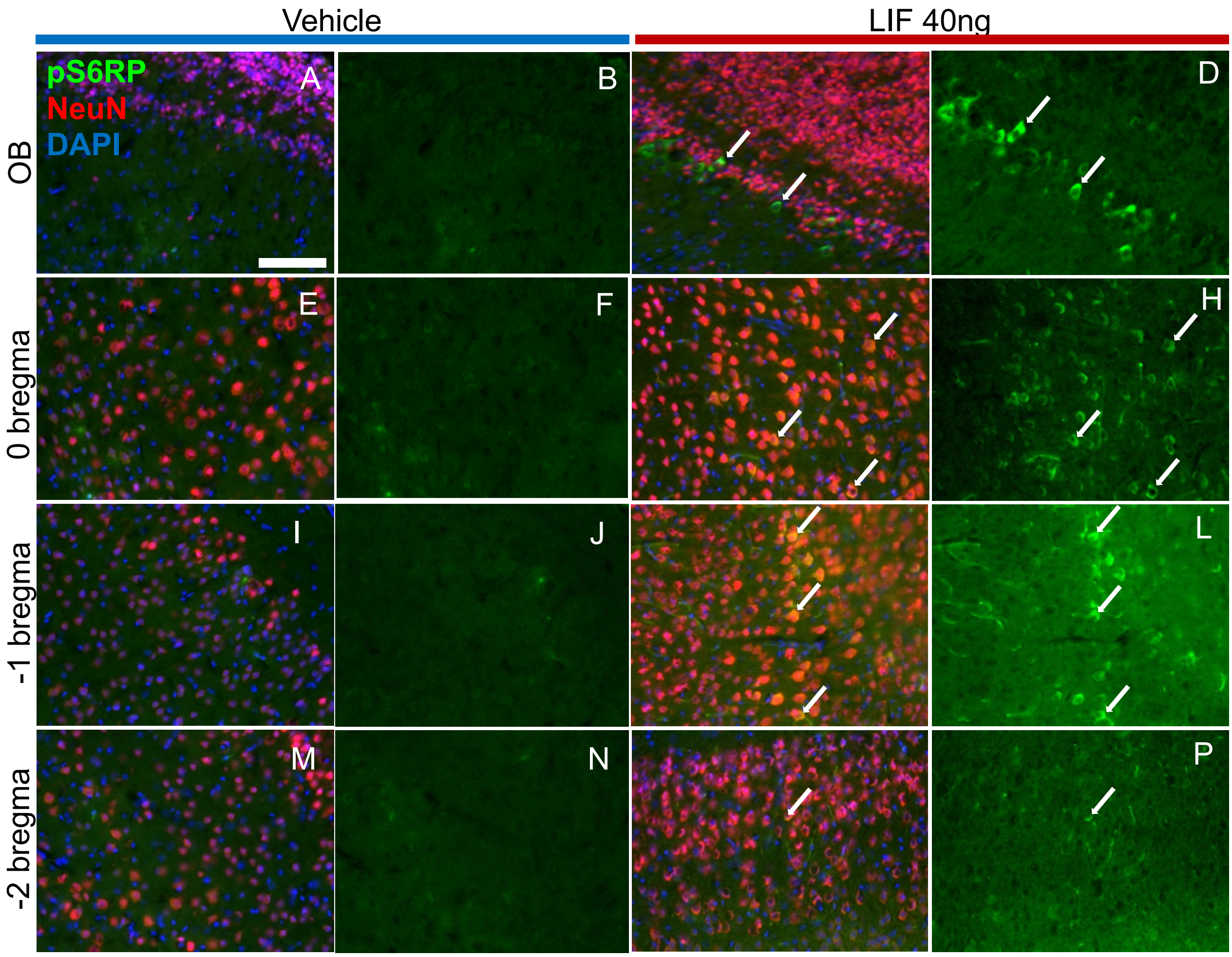

Fig S5. IN-LIF administration increases levels of phospho-S6 ribosomal protein (pS6RP). (A-P) Representative images at 20X magnification of pS6RP (green) co-labeled with neuronal marker NeuN (red) in sagittal sections of P18 WT CD-1 mice treated with IN-vehicle or 40ng IN-LIF for 30mins. Images were obtained from the olfactory bulb (OB) and neocortex at 0, -1 and -2 bregma. Scale bar = 50µm.
