# Supplementary material for: Intranasal Leukemia Inhibitory Factor Attenuates Gliosis and Axonal Injury and Improves Sensorimotor Function After a Mild Pediatric Traumatic Brain Injury
Source: Neurotrauma Rep. 2023 Apr 11;4(1):236–50. doi: 10.1089/neur.2021.0075 (PMC10122240; doi:10.1089/neur.2021.0075)
Supplement: Supplemental data [file Suppl_FigS6.pdf]

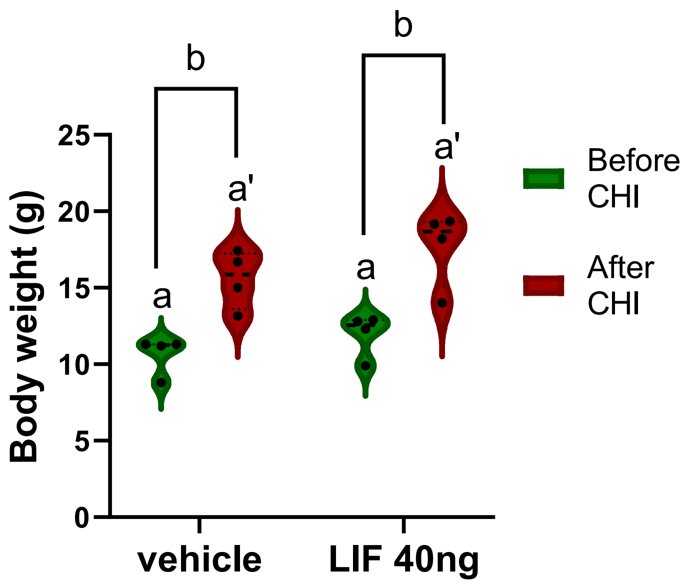

Fig S6. Acute IN-LIF dosage and regimen does not affect weight gain in pediatric mice after injury. Body weight of mice in grams before CHI (P18) and after CHI (P23). Body weight increased in both groups over time (b, F (1, 6) = 201.6, $p<0.0001$) but treatment did not have significant effect on weight gain (a and a', F (1, 6) = 1.928, p=0.2144). There was no interaction between treatment and time factors (F (1, 6) = 1.086, p=0.3375) by 2-way repeated measures ANOVA followed by Sidak's multiple comparisons test.
