# Supplementary material for: Intranasal Leukemia Inhibitory Factor Attenuates Gliosis and Axonal Injury and Improves Sensorimotor Function After a Mild Pediatric Traumatic Brain Injury
Source: Neurotrauma Rep. 2023 Apr 11;4(1):236–50. doi: 10.1089/neur.2021.0075 (PMC10122240; doi:10.1089/neur.2021.0075)
Supplement: Supplemental data [file Suppl_FigS7.pdf]

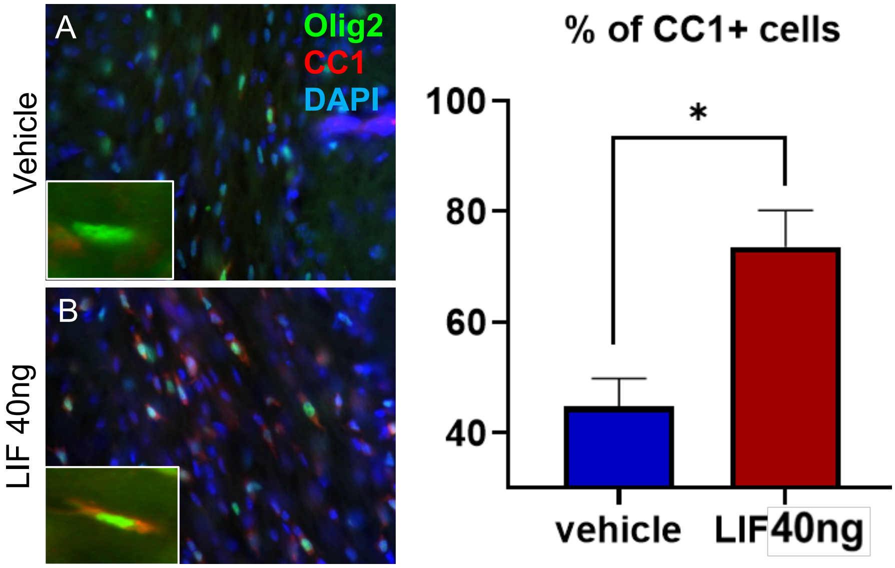

Fig S7. Subacute LIF Rx induces maturation of oligodendrocyte precursor cells. Mice received mTBI at P18 and IN LIF was initiated days later (P21**)** and administered twice daily for 3 days. Mice were perfused for histology on P26. **(A,B)** Representative images of oligodendrocytes (OLs) stained for OL nuclear protein Olig2 (green) and a marker of mature OL CC1 (red) at x dpi (x days after. Insets are higher power images of individual cells. **(C)** Percentage of mature CC1+ OLs of total Olig2+ OLs (*$p<0.05$, n=3 by unpaired, two-tailed t test).
